# 

**Published:** 1900-02

**Authors:** 


### CONTENTS.

ORIGINAL COMMUNICATIONS.

Interstitial Gingivitis due to Autointoxication. By EUGENE S. TALBOT, M.D., D.D.S. . . . . . . . 73
Influences that retard Evolution in Dental Science. By DR. S. B. PALMER, Syracuse, New York . . . . . . . . 84
Comparative Odontography. By A. H. THOMPSON, D.D.S. . . . . . . . . 102

ABSTRACTS AND TRANSLATIONS.

Gomphosis, A Barrier that cannot be swept Away. By B. H. CATCHING, D.D.S., Atlanta, Ga. . . . . . . . . 113

REPORTS OF SOCIETY MEETINGS.

National Dental Association . . . . . . . . 116
Massachusetts Dental Society . . . . . . . . 123

EDITORIAL.

Dental Educational Methods . . . . . . . . 134
Dentists in the Army . . . . . . . . 137

DOMESTIC CORRESPONDENCE.

Dentine Plastique du Docteur Klein. By I. B. DAVENPORT, M.D. . . . . . . 138

BIBLIOGRAPHY . . . . . . . . 141

OBITUARY.

Stephen Thomas Beale, M.D., D.D.S. . . . . . . . . 143

